# Sintering Behaviour of Waste Olivine and Olivine/Alumina Blends

**DOI:** 10.3390/ma7064773

**Published:** 2014-06-20

**Authors:** Erika Furlani, Eleonora Aneggi, Stefano Maschio

**Affiliations:** Department of Chemistry, Physics and Environment, University of Udine (UNIUD), Via del Cotonificio 108, I-33100 Udine, Italy; E-Mails: eleonora.aneggi@uniud.it (E.A.); stef.maschio@uniud.it (S.M.)

**Keywords:** olivine, alumina, sintering, crystal phases, microstructure

## Abstract

The sintering behaviour of several green compacts made with olivine or olivine/alumina powder blends has been examined. To this goal, powders were attrition milled, uniaxially pressed into specimens and air sintered at temperatures ranging from 1100 to 1300 °C. The resulting samples were characterized by water absorption, shrinkage, phase composition and density. Compositions containing 5%, 10% and 20% Al_2_O_3_ have a sintering behaviour similar to that of olivine alone, reaching low residual porosity when fired at 1300 °C. Conversely, the composition containing 40% Al_2_O_3_ displays an almost flat shrinkage profile and maintains high residual porosity in the examined temperature range.

## 1. Introduction

Olivine (O) sand is a natural raw material used by foundries to create moulds or cores particularly for casting manganese containing steels and is preferred to quartz sand as it minimizes the reactions between the liquid metal and the sand particles [1].

However, the ideal foundry sand particles should have a sub angular shape in order to allow the individual grains to interlock and form good moulds providing also the necessary pore spaces for superheated gases to escape without breaking the mould during the casting process. It follows that sand particle size affects the mould permeability and the finishing of the casted part. A suitable grain size distribution for foundry sand is centred on sieve 70 (212 μm ) by U.S. Standard [2], with very little sand being retained on sieve sizes lower than 30 (590 μm) or greater than 140 (105 μm). As a consequence, all commercially available sands supplied to a foundry, must be de-pulverized in order to cut off their finest fraction, before being used for the moulds preparation. This fine sand fraction turns into a by-product, although not a real waste, and ideally would need to be used in any other industrial process rather than an eventual landfill disposal. Olivine fine powder represents therefore an out of the ordinary by-product which requires a possible valuable industrial application.

The possible use of Mg containing silicates such as forsterite or cordierite in the ceramic industry is widely documented in the literature [3,4,5,6,7], but that of olivine or O containing powders is presently confined to the production of some special refractory bricks [8,9] or particular glazes for tiles [10].

On the other hand, straight O powder or enriched by other metal elements could be used as a catalyst for fluidized bed reactors (FBR) in biomass combustion. This option is presently accepted as a possible viable application of O and it is supported by an extended literature [11,12,13,14,15,16,17,18,19,20,21,22,23,24,25,26,27,28,29]. For such application, O powders must maintain high catalytic properties at temperatures ranging from 750 to more than 1000 °C as those are the conditions generally used for biomass combustion [11,12,13,14,15,16,17,18,19,20,21,22,23,24,25,26,27,28,29,30,31,32].

O is a natural material which can be mined from ores. It is generally known that powders obtained by grinding natural materials have low specific surface area and also the waste fine fraction derived from the depulverization of coarse olivine sand has itself almost a low specific surface area. It is generally accepted that its eventual wet or dry milling does not enhance it significantly. The addition of a large surface area alumina powder could lead to the preparation of blends with larger surface area with respect to olivine alone and therefore would perform better in heterogeneous catalytic reactions as efficient support or as real catalytic material at any temperature.

The present research aims to study the sintering behaviour of O fine powders alone or mixed, in different proportions, with a high grade alumina (A). The study also highlights phases and microstructures which take place after a thermal treatment at high temperature. With these goals in mind, powders of each composition, pressed into several specimens, were fired at different temperatures for 1 h, then shrinkage and water absorption were measured in order to build up their sintering curves; apparent density, crystal phases and microstructures were investigated as a function of the sintering cycle.

## 2. Results and Discussion

**Figure 1:**
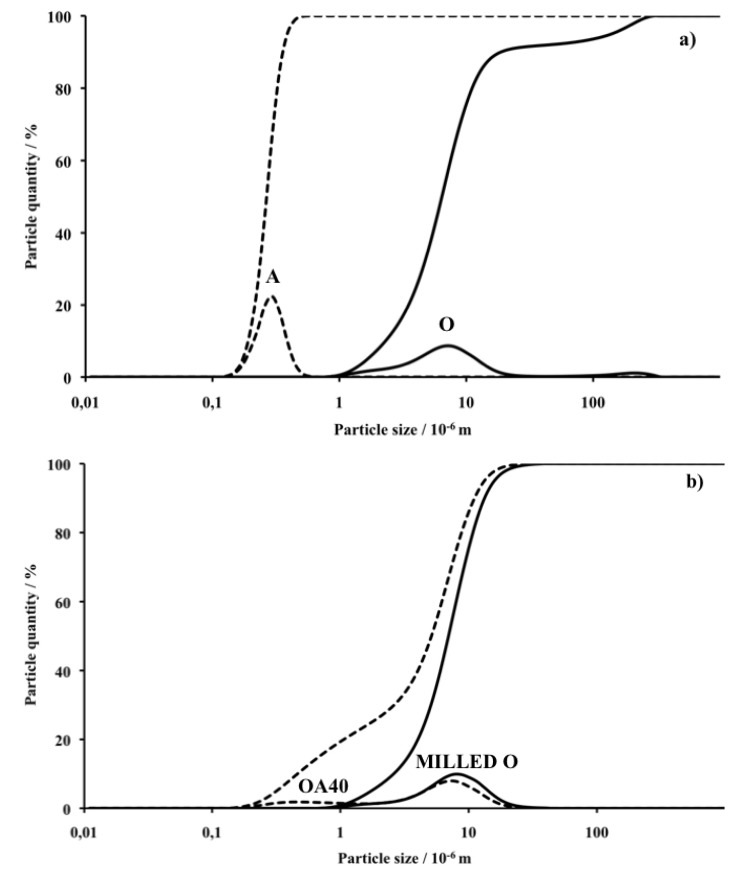
Particle size distribution (PSD) and accumulated quantity of the powders before and after the milling process: (**a**) as received olivine (plain line) and α-alumina (dashed line); (**b**) the blend OA40 (dashed line) and olivine (plain line) after milling. Diagrams are represented with logarithmic abscissa.

Figure 1a shows the PSD curves of the as received O and A powders whereas Figure 1b reports the PSD of milled O alone and that of the blend OA40 which was selected among the powders blends after the milling procedure. It can be observed that O displays a bimodal PSD with a large peak centred at about 10 μm and a small one centred at 200 μm. In order to improve powder densification during subsequent pressing and sintering, such PSD must be optimized in order to transform the raw product into a powder with particles distributed within a narrow and possibly micronic range [33,34,35]. Alumina powder displays a monomodal PSD with a single narrow peak centred on 280 nm with inferior particles limit of 110 and superior of 600 nm, therefore in line with the above described characteristics. The PSD of the milled O alone shows the presence of a monomodal distribution of particles with the maximum concentration at 7 μm whereas the curve representative of the blend OA40 shows that the presence of A modifies the curve of O giving rise to a shoulder at 280 nm which corresponds to the size of most A particles. Definitively it is observed that the attrition milling: (I) reduces particles size to less than 30 μm; (II) the milled blends of powder display PSD curves that progressively change their shape from the one of O alone to that of the blend OA40 which highlight the presence of a second component with a larger surface area. As a consequence of the attrition milling which reduces particle size of the starting powders to less than 30 μm, it was assumed that crystal phases, microstructures and apparent density of the sintered materials are almost independent of the powders PSD.

As previously reported [36,37] chemical analysis revealed that O contains high quantities of SiO_2_, MgO, Fe_2_O_3_, minor quantities of Al_2_O_3_, CaO and small fractions of NiO, Cr_2_O_3_ and MnO; A is a high grade product and its chemical analysis is not reported in the present paper. The XRD investigation revealed that the “as received” O powder mainly contains forsterite (Mg_2_SiO_4_) (PDF 01-079-1210), enstatite (MgSiO_3_) (PDF 00-019-0768) and quartz (PDF 01-083-2465) as minor phase, which has been blended with α-Al_2_O_3_.

**Figure 2:**
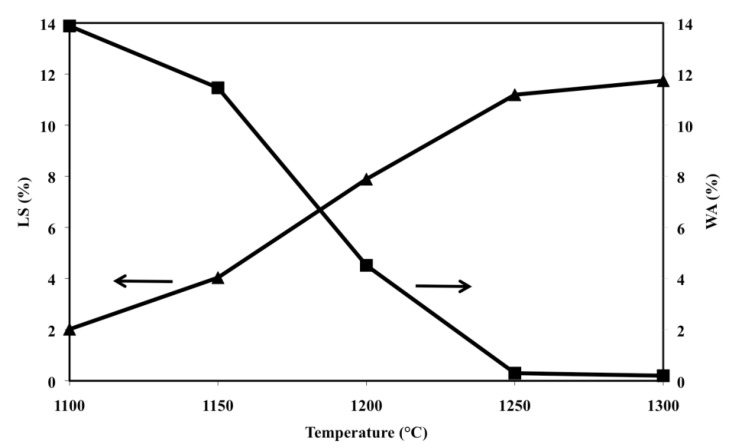
Linear shrinkage (LS) and water absorption (WA), as a function of firing temperature of samples made of O alone (LS: ▲; WA: ■).

The sintering behaviour of the various compositions can be documented by materials shrinkage and water absorption after the firing process; such trends, for samples made with O alone, are included (Figure 2) in a previous work [36] but have been reported also in the present paper for easy comprehension by the reader. Olivine samples, when fired at temperatures greater than 1300 °C, soften and lose their shape (due to the formation of liquid phase) thus shrinkage cannot be clearly measured and its representative line ends at 1300 °C. Below 1300 °C the line has the shape of an elongated S characterized by two major slope changes, the first at 1150 °C, the second at 1250 °C. The complementary water absorption line is similar to a taut Z, with two major slope changes which fall at the same temperatures (1150 and 1250 °C) reported above. It must be also pointed out that samples fired at temperatures equal or greater than 1250 °C display water absorption close to zero.

**Figure 3:**
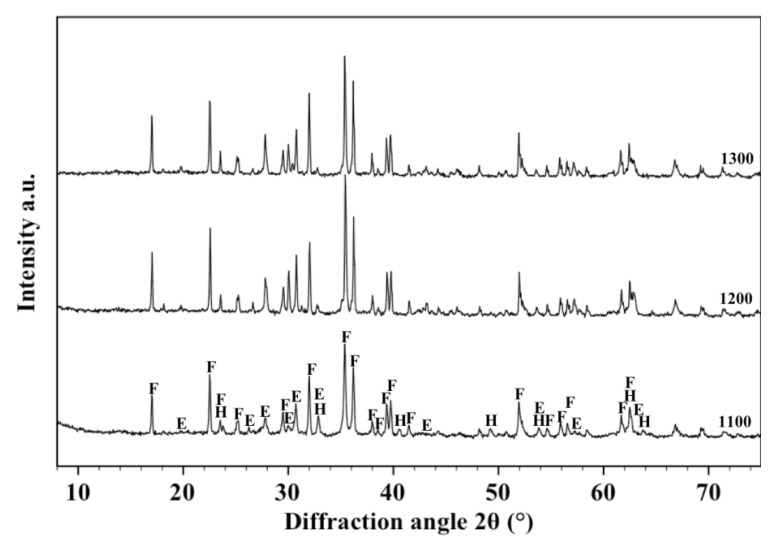
X-ray diffraction patterns between 10° and 70° acquired on the free surface of samples made with O alone fired at 1100, 1200 and 1300 °C. Phases are identified by the following symbols: (E) = enstatite, (F) = forsterite and (H) = hematite.

Figure 3 shows the XRD patterns acquired on the samples fired respectively at 1100, 1200 and 1300 °C. It can be observed that the starting crystallographic composition of olivine progressively changes with the firing temperature so that hematite, enstatite and forsterite can be detected after a thermal treatment at 1100 °C, but only enstatite and forsterite at higher temperatures. It could be deduced that the two slope changes observed in the shrinkage and water absorption lines are related to the disappearance of hematite which melts into the liquid phase whose quantity increases with temperature, favouring materials densification by liquid phase sintering [38,39,40]. These results are sufficiently in agreement with those reported by Michel *et al.* [41] who detected the presence of almost similar (but not exactly the same) phases after thermal treatment of O powders at 1400 °C. Such discrepancies are reasonably explained due to the different chemical/crystallographic composition of the starting powders and to the different experimental procedure followed in the two works.

**Figure 4:**
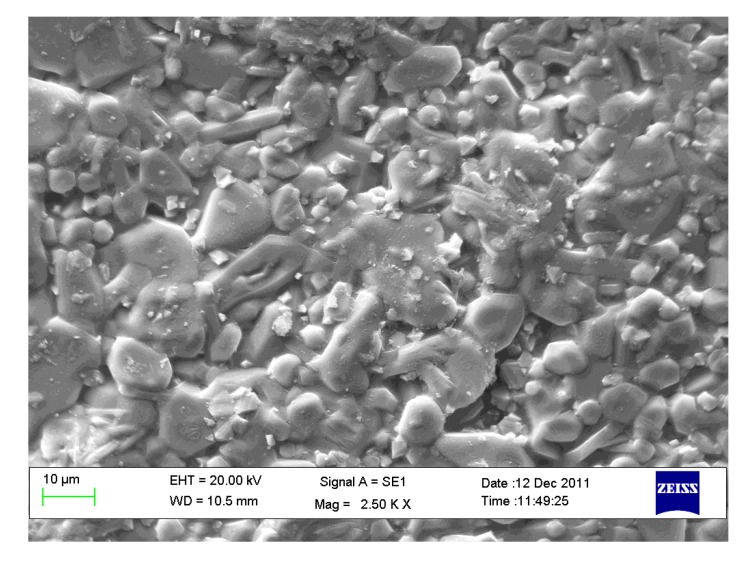
Scanning Electron Microscope (SEM) micrography (2500×) of the surface of sample made with olivine fired at 1300 °C.

Figure 4 shows the microstructure of a sample, made with O alone, after the sintering cycle at 1300 °C for 1 h. The presence of mainly equiaxial grains of size around 10 μm is documented together with the vitreous phase which can be clearly identified where grain boundaries are not well defined. The apparent density measured on the above material is 2.96 g/cm^3^, whereas the literature theoretical density of enstatite and forsterite is 3.2 g/cm^3^[42]. If this value is assumed as the theoretical density of this composition, the apparent density/theoretical density ratio (relative density) leads to a total porosity of 7.5%; since its water absorption is about zero it could be deduced that there is almost no open porosity which is confirmed by the SEM (Scanning Electron Microscope) analysis. Thus, it could be assumed that, together with the vitreous phase, samples also contain a non-negligible close porosity which cannot be accurately quantified by this indirect method.

The XRD patterns of OA5 samples fired at 1100, 1200 and 1300 °C are displayed in Figure 6a. It can be observed that, at 1100 °C, they contain forsterite (64%), Fe-rich enstatite (28%) (PDF 01-071-1163), hematite (4%) (PDF 01-089-0599) and α-alumina (4%), but, in agreement with the results of other researchers [43,44,45], at higher temperatures alumina reacts with forsterite to form the MgOAl_2_O_3_ spinel phase (PDF 01-075-1798) which raises from 10%, after a thermal treatment at 1200 °C, to 15% after a thermal treatment at 1300 °C. Conversely, the quantity of forsterite decreases from 64% to 60% and enstatite from 28% to 25%. Crystallographic data and densities of all the samples fired at 1300 °C are summarized in Table 1.

**Table 1:** Phases, corresponding percentage (wt%), approximate theoretical density (g/cm^3^), apparent density and relative density of the materials fired 1 h at 1300 °C.

Sample	Phase	wt%	Theoretical density (g/cm^3^)	Apparent density (g/cm^3^)	Relative density (%)
OA5	Forsterite	60	3.28	3.05	93.0
	Fe-Enstatite	25			
	Spinel	15			
OA10	Forsterite	52	3.28	3.05	93.0
	Fe-Enstatite	33			
	Spinel	15			
OA20	Forsterite	37	3.29	3.05	92.7
	Fe-Enstatite	36			
	Spinel	23			
	Cordierite	4			
OA40	Forsterite	19	3.33	3.01	90.4
	Fe-Enstatite	36			
	Spinel	32			
	Cordierite	10			
	α-Alumina	3			

## 2.1. Compositions OA5 and OA10

**Figure 5:**
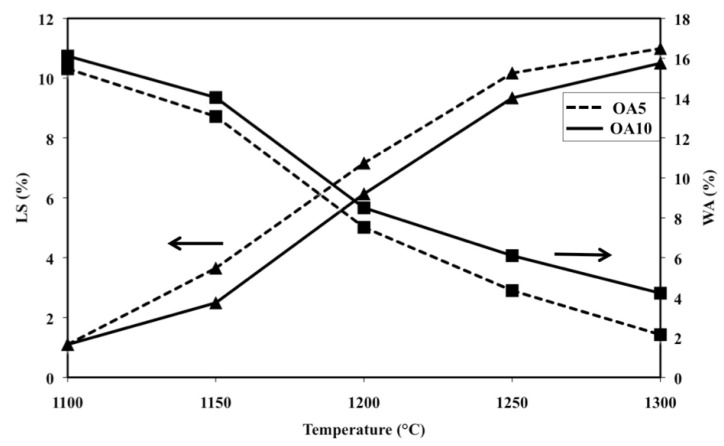
Linear shrinkage (LS) and water absorption (WA) as a function of firing temperature of samples OA5 and OA10 (LS: ▲; WA: ■).

The addition of a small amount of A does not strongly modify the sintering behaviour of O. Such a statement is confirmed by Figure 5 which shows shrinkage and water absorption versus temperature of the specimens OA5 and OA10. It can be observed that such compositions display similar but not same trends since the shrinkage and water absorption lines run almost parallel. OA5 performs better than OA10 because the shrinkage and water absorption values of the former are better than those of the latter at every temperature.

**Figure 6:**
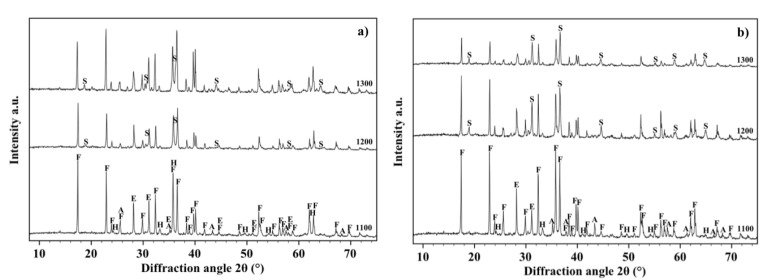
X-ray diffraction patterns between 10° and 70° acquired on the free surface of samples OA5 (**a**) and OA10 (**b**) fired at 1100, 1200 and 1300 °C. Phases are identified by the following symbols: (E) = enstatite, (F) = forsterite, (H) = hematite, (A) = alumina and (S) = spinel.

**Figure 7:**
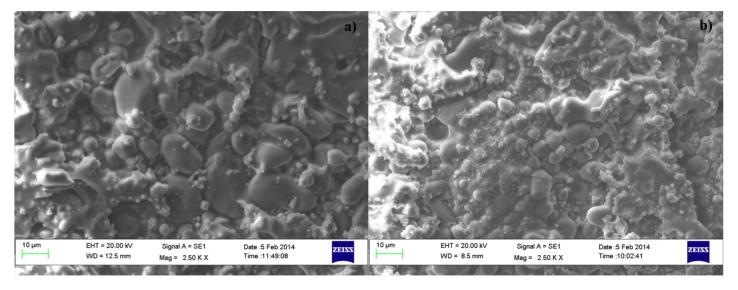
SEM micrographies (2500×) of the fired surface of samples with composition: OA5 (**a**) and OA10 (**b**).

Figure 6b shows the XRD patterns, acquired on the free surface of OA10 samples, after firing at 1100, 1200 and 1300 °C. It can be observed that most phases identified in materials with composition OA5 can be revealed also in composition OA10. In fact, forsterite, alumina, enstatite and hematite have been detected in samples fired at 1100 °C but, after a thermal treatment at 1200 °C, forsterite reacts with alumina to form a spinel phase and its quantity decreases from 65% to 54%. At the same time, enstatite (PDF 01-072-1507) reacts with hematite to form a Fe-rich enstatite (PDF 01-071-1163) and its quantity increases from 22% to 32% after thermal treatment at 1200 °C. The amount of forsterite (52%), spinel (15%) and Fe-rich enstatite (33%) does not significantly change in samples fired at 1300 °C. In each composition, the amount of liquid phase progressively increases with temperature enabling shrinkage to fully dense samples [38,39,40] as is demonstrated by Figure 5. It can also be observed in Figure 7a,b which are two SEM micrographs of the “as fired” surface of samples with composition OA5 and OA10 respectively, after the cycle at 1300 °C. Each material shows no open porosity and the presence of a certain quantity of vitreous phase which is evident at most of the grain boundaries. However, if the quantity of glass is assumed negligible with respect to the amount of crystalline phases, their theoretical density can be calculated using XRD data which leads to the value of 3.28 g/cm^3^ for both compositions which also have the same apparent density (3.05 g/cm^3^) and relative density (93.0%). As discussed for O samples, also of these compositions, the presence of non-visible close porosity must be hypothesized. Figure 7a,b also show, in each material, two typologies of grains: one rounded with size ranging from less than 5 μm to more than 10 μm, the other, of submicronic size, well faceted which seems to emerge from the surface in light shade. It can also be remarked that on increasing the amount of A, the size of the polycrystalline grains becomes smaller.

## 2.2. Composition OA20 and OA40

**Figure 8:**
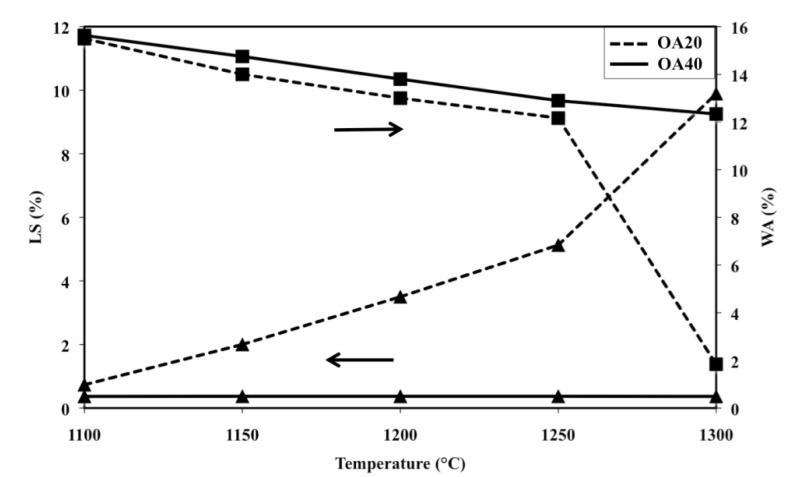
Linear shrinkage (LS) and water absorption (WA) as a function of firing temperature of samples OA20 and OA40 (LS: ▲; WA: ■).

Figure 8 shows shrinkage and water absorption of samples OA20 and OA40 in the range 1100–1300 °C. It can be observed that the water absorption of samples with composition OA20 displays an almost linear decreasing trend between 1100 and 1250 °C, which changes slope for higher temperatures. Simultaneously, shrinkage shows an almost linear increasing trend with values below 5% up to 1250 °C, but for higher temperatures the slope of the line changes and reaches the value of 10% after a thermal treatment at 1300 °C.

Linear shrinkage versus temperature of composition OA40 can be represented by an almost flat line as a result of values that remain below 1% at any temperature; on the other hand water absorption shows an almost decreasing linear trend which starts from about 16% after the firing cycle at 1100 °C and ends at 12% after the thermal treatment at 1300 °C.

**Figure 9:**
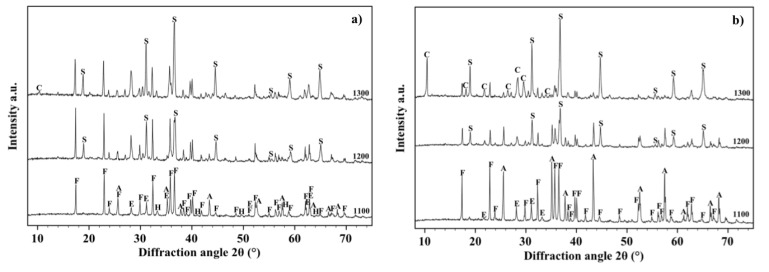
X-ray diffraction patterns between 10° and 70° acquired on the free surface of samples OA20 (**a**) and OA40 (**b**) fired at 1100, 1200 and 1300 °C. Phases are identified by the following symbols: (E) = enstatite, (F) = forsterite, (H) = hematite, (A) = alumina, (S) = spinel and (C) = cordierite.

Figure 9a shows the XRD patterns, acquired on the free surface of samples with composition OA20, after sintering at 1100, 1200 and 1300 °C. It can be observed that forsterite (61%), alumina (18%), enstatite (17%) and hematite (4%) are detected after firing at 1100 °C. If the amount of the liquid phase is assumed negligible, it could be argued that in samples fired at 1200 °C hematite totally dissolves into the Fe-rich enstatite phase (33%) and alumina completely reacts with forsterite to form the spinel phase (10%). The firing cycle at 1300 °C causes the formation of a small amount of α-cordierite (4%) (PDF 01-089-1485) due to the reaction between forsterite and the liquid phase: forsterite is reduced to 37%, spinel rises up to 23% whereas enstatite remains almost constant (36%). The μ-cordierite was not revealed by the present investigation reasonably due to the presence of an excess of MgO [42,45,46].

Figure 9b shows the XRD patterns acquired on the free surface of samples with composition OA40 after sintering at 1100, 1200 and 1300 °C. In detail it has been observed that forsterite (43%), alumina (35%) and the Fe-rich enstatite (22%) phase were detected after the sintering cycle at 1100 °C; specimens fired at 1200 °C led to identify forsterite (31%), spinel (21%), alumina (24%) and the Fe-rich enstatite phase (24%); samples fired at 1300 °C contain forsterite (19%), the Fe-rich enstatite phase (35%), spinel (33%), 10% of cordierite and a residual 3% of alumina. Finally, in the materials with composition OA40 two high temperature reactions between alumina and forsterite take place: The former occurs between 1100 and 1200 °C and leads to the formation of spinel, the latter occurs above 1200 °C and leads to cordierite crystallization coupled to the increase of the spinel quantity.

**Figure 10:**
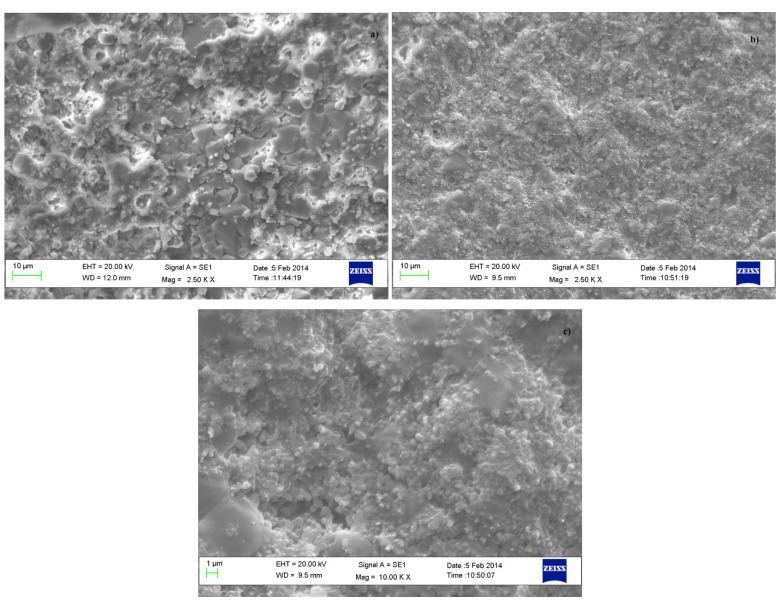
SEM micrographies (2500×) of the fired surface of samples with composition: OA20 (**a**) and OA40 (**b**). (**c**) shows an enlarged detail of Figure 10b (10,000×).

Figure 10a and b are two SEM micrographs of the as fired surface of samples with composition OA20 and OA40 respectively, after the cycle at 1300 °C. It can be observed that the specimen with composition OA20 shows no large sized open porosity, but a certain quantity of vitreous phase, lower than that observed in compositions OA5 and OA10; at the same time, two typologies of grains, similar to those already observed in composition OA5 and OA10 are visible. Conversely, the sample with composition OA40 does not contain grains of size larger than 1 μm. Figure 10c is a detail of Figure 10b; it is reported for clarity and confirms the above statement. With this in mind, if the amount of the glassy phase is assumed negligible with respect to the crystalline, the theoretical density, calculated using XRD data, is 3.29 g/cm^3^ for OA20 and 3.33 g/cm^3^ for OA40. The apparent density of OA20 after the firing cycle at 1300 °C is 3.05 g/cm^3^, whereas that of OA40 is 3.01 g/cm^3^ which lead to the relative densities of 92.7% and 90.4% respectively. It means that material OA20 should contain a certain close porosity, but not open porosity, whereas material OA40, which also shows a moderate water absorption, should contain open as well as close porosity. The unexpected sintering behaviour of composition OA40 is reasonably explained due to the high content of alumina which leads to the formation of a high quantity of the spinel phase. The reaction takes place in the presence of a moderate amount of liquid phase. The synergic effect of alumina, spinel and limited amount of liquid phases gives rise to several solubilisation-reprecipitation processes which lead to fine grained, highly porous materials which also display low shrinkage on firing as has been previously reported also by other authors [47,48,49,50,51]. The above described phenomenon could be amplified by a possible overfiring effect typically enhanced by the presence of MgO which also could cause de-sintering and eventually bloating [6]. In addition, it is worth remarking that it has been observed that increasing the amount of alumina, also increases the amount of Fe-rich enstatite in most of the fired samples. This result is apparently in conflict with the chemical composition of the materials, but can be explained by taking into account their microstructure. It is generally accepted that iron oxide easily melts into an eventual high temperature liquid phase, decreasing its viscosity. With the present SEM investigation, it was observed that on increasing the amount of alumina, the amount of liquid phase decreases. The original quantity of iron oxide introduced by olivine is shared into vitreous and polycrystalline phases; if the amount of the former decreases then Fe must be mainly confined into the Fe-containing polycrystalline phases, which, in our materials, are represented by enstatite or Fe-rich enstatite.

Speculatively, composition OA40 could find possible applications where powder sintering is minimized, for instance in catalysis, or where a high residual porosity is one of the most required characteristics such as refractory bricks [8,9].

## 3. Materials and Methods

The O powder used in the present work is a by-product of a foundry process dedicated to the production of a high manganese steel and contains particles of sizes smaller than 500 μm. It was used alone or blended with 5, 10, 20 and 40 wt% of a high grade alumina (Sumitomo AK30, Tokyo, Japan). Symbols used for sample identification are respectively O, OA5, OA10, OA20 and OA40.

The chemical composition of the above raw materials, obtained by a Spectro Mass 2000 Induced Coupled Plasma (ICP) mass spectrometer, is reported in Table 2 which also displays the lost on ignition (LOI) after a thermal treatment at 1000 °C for 2 h.

O alone and all the blends (70 g of powder for each preparation) were homogenized by attrition milling for 1 h in a home-made instrument. Milling parameters were as follows: High-density nylon container (volume = 750 mL); 500 g of 99 wt% alumina balls (diameter = 6–8 mm); 150 mL of distilled water; 300 cycles min^−1^. At the end of the milling process, slurries were oven dried for 24 h at 80 °C.

After milling, Particle Size Distribution (PSD) was evaluated using a Horiba LA950 laser scattering PSD analyser (Horiba Ltd, Kyoto, Japan): analyses were made in water after a 3 min sonication time. For clarity of comprehension, curves are represented with logarithmic abscissa, as is commonly done for the presentation of this type of result.

**Table 2:** Composition (wt%) and lost on ignition (LOI) (%) of Olivine (O) used as starting material in the present study.

Component	Olivine	Component	Olivine
SiO_2_	41.35	TiO_2_	< 0.01
Al_2_O_3_	0.96	NiO	0.27
CaO	1.08	MnO	0.06
MgO	45.65	P_2_O_5_	< 0.01
Na_2_O	< 0.01	SO_4_^=^	< 0.01
K_2_O	< 0.01	Cl^-^	< 0.01
FeO	6.61	Undetermined	0.73
Cr_2_O_3_	0.19	LOI	3.10

Dried powders were sieved (200 μm ~70 mesh) and uniaxially pressed at 100 MPa into cylindrical specimens (Φ = 27 mm, *h* = 4.5 mm).

Sintering experiments were performed in air, by an electric muffle, at several temperatures ranging from 1100 to 1400 °C with intervals of 50 °C using heating and cooling rates of 10 °C min^−1^ and a dwell time of 1 h.

Shrinkage on firing was evaluated, by a caliper, along the diameter (27 mm on green specimens) using the ratio (Φ_0_ − Φ_1_)/Φ_0_ (subscripts 0 and 1 refer to the sample dimensions before and after the sintering). Apparent density of the sintered specimens was determined by the Archimedes method. Water absorption was determined following the UNI EN ISO 10545-3:2000; in line with this standard, fired samples were first weighed in air (*W*_1_), then placed in a covered beaker and boiled in water for 2 h. After boiling, samples were cooled in water to room temperature, dried with a cloth and weighed again (*W*_2_). Water absorption was evaluated using the formula: *W* (%) = 100 [(*W*_2_ − *W*_1_)/*W*_1_].

Crystal phases were investigated by X-ray diffraction (XRD) analysis which was carried out on a Philips X’pert Pro Detector X’celerator (Philips, Eindhoven, The Netherland) operated at 40 kV and 40 mA using Ni-filtered Cu-Kα radiation. Spectra were collected using a step size of 0.02° and a counting time of 15 s per angular abscissa in the range 10°–70°. The Philips X’Pert HighScore software was used for phase identification the semi-quantitative evaluation being performed following the RIR method [52]. Microstructures were examined by an Zeiss EVO40 Scanning Electron Microscope (SEM) (Carl Zeiss GmbH, Göttingen, Germany).

## 4. Conclusions

The present research deals with production and characterization of ceramics containing olivine (O) powder and a high grade alumina (A). O-A blends were mixed by wet attrition milling, dried, sieved, pressed into specimens and fired at several temperatures in the range 1100–1300 °C for 1 h.

The sintering experiments demonstrated that:

(1) compositions containing 5, 10 and 20 wt% of A display a sintering behaviour similar to that of O alone, whereas samples containing 40% A show an almost flat profile of the shrinkage line in the investigated temperature range;
(2) compositions containing 5% and 10% A, when fired at temperature above 1200 °C, show evidence of the spinel phase which coexists with forsterite, a Fe-rich enstatite and the vitreous phase;
(3) compositions containing 20% and 40% A, when fired at temperatures above 1200 °C, show evidence of a limited quantity of α-cordierite which coexists with the spinel phase, forsterite and a Fe-rich enstatite;
(4) the grain size of materials fired at 1300 °C decreases on increasing the quantity of A and is reduced below 1 μm in materials containing 40% of A, probably due to the presence of a high amount of spinel phase which is formed during the sintering process.
